# A comparison of clinical paediatric guidelines for hypotension with population-based lower centiles: a systematic review

**DOI:** 10.1186/s13054-019-2653-9

**Published:** 2019-11-27

**Authors:** Nienke N. Hagedoorn, Joany M. Zachariasse, Henriette A. Moll

**Affiliations:** Department of Paediatrics, Room Sp 1540, Erasmus MC-Sophia Children’s Hospital, University Medical Centre Rotterdam, PO Box 2060, 3000 CB Rotterdam, The Netherlands

**Keywords:** Vital signs, Hypotension, Percentiles, Reference values

## Abstract

**Background:**

Different definitions exist for hypotension in children. In this study, we aim to identify evidence-based reference values for low blood pressure and to compare these with existing definitions for systolic hypotension.

**Methods:**

We searched online databases until February 2019 (including MEDLINE, EMBASE, Web of Science) using a comprehensive search strategy to identify studies that defined age-related centiles (first to fifth centile) for non-invasive systolic blood pressure in healthy children < 18 years. Existing cut-offs for hypotension were identified in international guidelines and textbooks. The age-related centiles and clinical cut-offs were compared and visualized using step charts.

**Results:**

Fourteen studies with population-based centiles were selected, of which 2 addressed children < 1 year. Values for the fifth centile differed 8 to 17 mmHg for age. We identified 13 clinical cut-offs of which only 5 reported accurate references. Age-related cut-offs for hypotension showed large variability (ranging from 15 to 30 mmHg). The clinical cut-offs varied in agreement with the low centiles. The definition from Paediatric Advanced Life Support agreed well for children < 12 years but was below the fifth centiles for children > 12 years. For children > 12 years, the definition of Parshuram’s early warning score agreed well, but the Advanced Paediatric Life Support definition was above the fifth centiles.

**Conclusions:**

The different clinical guidelines for low blood pressure show large variability and low to moderate agreement with population-based lower centiles. For children < 12 years, the Paediatric Advanced Life Support definition fits best but it underestimates hypotension in older children. For children > 12 years, the Advanced Paediatric Life Support overestimates hypotension but Parshuram’s cut-off for hypotension in the early warning score agrees well. Future studies should focus on developing reference values for hypotension for acutely ill children.

## Introduction

Vital signs are important in the recognition of acutely ill children. One parameter associated with serious illness is hypotension [1–3]. Because normal blood pressure values vary with age, accurate age-related reference values are needed to correctly identify hypotension in children and guide interventions.

Blood pressure can be measured by invasive, oscillometric and auscultatory methods. In addition, various outcome measures for blood pressure exist such as mean arterial pressure, and diastolic and systolic blood pressure. Paediatric guidelines propose different definitions of hypotension and in general use cut-off values of systolic blood pressure [4–6]. Although not based on evidence, several guidelines use the fifth percentile of systolic blood pressure in healthy children as cut-off for hypotension [4, 7, 8]. Moreover, it is unclear how well these guidelines discriminate between normal and low blood pressure. To date, no study has summarized the available evidence on reference values of low systolic blood pressure in children.

This study aims to identify population-based reference values for non-invasive low blood pressure in healthy children and to compare these with cut-offs for hypotension defined by existing paediatric guidelines.

## Methods

### Search strategy and selection of population-based studies

We systematically searched databases including MEDLINE, EMBASE and other databases (1950 to 14 February 2019) to identify primary studies that defined lower centiles for non-invasive systolic blood pressure measurement in healthy children (Additional file 1: detailed search strategy). Studies that were included were published in English, recorded blood pressure and defined age-related centiles for systolic blood pressure (first to fifth centile) on a minimum of 100 children aged < 18 years. Studies were excluded if populations involved children with underlying diseases, or studies reporting on premature neonates, measurements during anaesthesia, exercise or orthostasis. We excluded populations from low- and middle-income countries since factors influencing blood pressure levels, such as body composition and nutrition, are different compared to high-income countries [9]. We excluded abstracts, reviews and commentaries, and studies reporting on lower centiles solely derived from mathematical analysis. One researcher (NH) conducted the first selection, and two researchers (NH, JZ) independently conducted the second and third selection. Disagreements were discussed and agreed upon consensus or discussed with a third researcher (HM) for majority decision.

### Data extraction and analysis

For the selected studies, data were extracted by one researcher (NH) and included country, population, setting, sample size, age range, blood pressure measurement method and age-specific centiles (P1–P5). We included the centiles for non-overweight children and for the median height if blood pressure centile values were reported for different height categories. The age-specific fifth centiles were summarized using weighted medians and interquartile ranges for age categories which involved three or more studies. If sample sizes were only given for age ranges > 1 year, we estimated the sample size per age group by dividing the total sample size by the number of years.

### Quality assessment

No specific tool exists for quality assessment of observational studies [10]. The Quality Assessment of Diagnostic Accuracy Studies-2 checklist was the most appropriate to use for these observational studies [11]. This checklist covers risk of bias and applicability judgements on four domains: patient selection, index test, reference standard and flow and timing. For each question, studies were classified as high, low or unclear. Disagreements were agreed upon consensus.

### Cut-off values for hypotension from clinical guidelines

We selected a sample of clinical cut-offs for hypotension by consulting experts, well-known textbooks and resuscitation, emergency care and sepsis guidelines. Clinical cut-offs included recommended target values for hypotension defined by systolic blood pressure. For each clinical cut-off, we determined the presence of a literature reference and whether this reference agreed with the cut-off values. To compare clinical cut-offs with the population-based centiles identified in the literature, we plotted the age-specific fifth centile values in a step chart separate for boys and girls. Data analyses were performed in SPSS version 25.0 and R version 3.4.

## Results

### Population-based studies

**Fig. 1:**
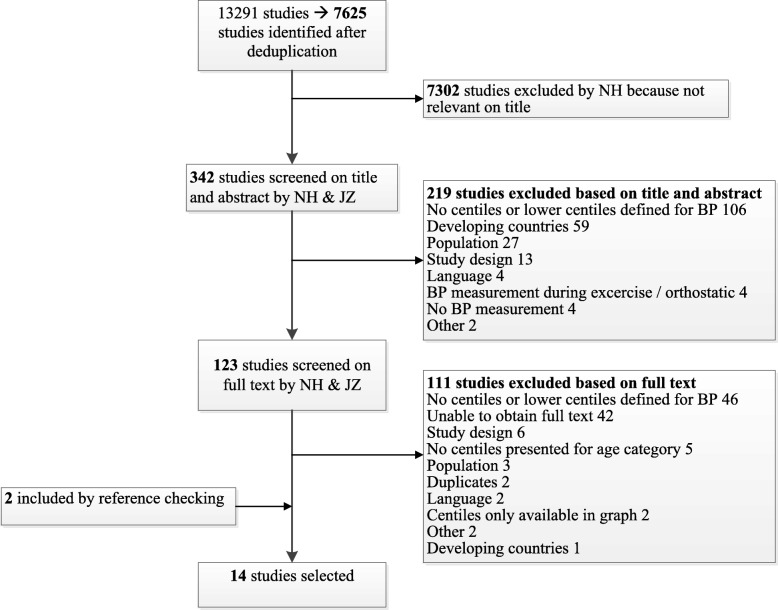
Study selection process. BP, blood pressure

**Fig. 2:**
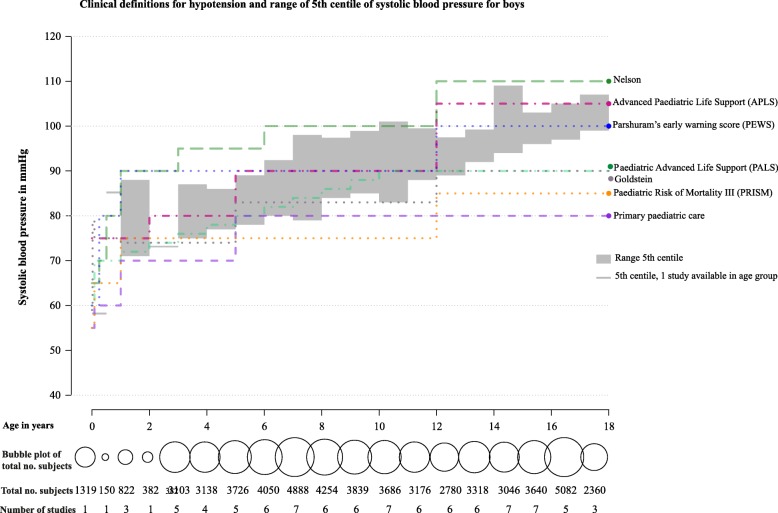
Clinical definitions for hypotension and range of fifth centile of systolic blood pressure for boys

Our systematic search identified 7625 studies. After the study selection process, we included 14 studies in the final selection that defined lower centiles for non-invasive systolic blood pressure measurement in healthy children (Fig. 1). The median sample size was 5362 (IQR 1760–11,940). Seven out of 14 studies used an automatic oscillometric device for blood pressure measurement. Two studies included children aged < 1 year (Table 1). Studies included populations from Europe (*n* = 8), North America (*n* = 3), Australia (*n* = 2) and Asia (*n* = 1). Four studies excluded overweight patients. For development of the centiles, 11 studies used the average of multiple blood pressure measurements and 3 studies used only the first measurement. Blood pressure centiles were stratified by gender (*n* = 12), height (*n* = 4), ethnicity (*n* = 1) and overweight vs non-overweight (*n* = 2). Studies most frequently reported the fifth centile (*n* = 13), in which the third centile (*n* = 2) and first centile (*n* = 3) were also reported separately. One study only reported the first and third centiles. The fifth centiles of the population-based studies showed variation ranging across the age groups from 7 to 17 mmHg for boys (Fig. 2) and 7 to 22 mmHg for girls (Additional file 2). Median values and interquartile ranges of the lower fifth centiles are provided in Additional files 3 and 4.

**Table 1:** Characteristics of included studies

Author	Country	Inclusion	Exclusion	Age range (years)	Setting	Sample size	Method of measurement	Defined BP centiles	Determinants of age-specified centiles	Measurement used for analysis	Main outcome
Antal et al. [12]	Hungary	Secondary school	Using antihypertensive medication	15–18	Community setting	6345	Oscill.	P3, P5	Sex	First measurement	Assessment of age- and gender-specific anthropometric parameters and blood pressure values
Barba et al. [13]	8 EU countries	Non-overweight children	Overweight	2–10.9	Unspecified	13,547	Oscill.	P1, P3	Sex, height	Mean of first and second measurement	Provide oscillometric blood pressure reference values
Blake et al. [14]	Australia	Cohort from a tertiary perinatal centre. Follow-up at age 1, 3 and 6 years	x	1–6	Unspecified	2876	Oscill.	P5	Sex	Mean of two measurements	To develop age- and gender-specific reference ranges for BP
Grajda et al. [15]	Poland	Healthy pre-school children	Congenital, chronic or acute disorders and medication affecting growth or BP levels	3–6	Community setting	4378	Oscill.	P1, P5	Sex, height	Mean of second and third measurement	To develop age- and gender-specific ranges for BP in pre-school children
Hediger et al. [16]	USA	Black adolescents	x	11–17	Unspecified	621	Auscul.	P5	Sex	Mean of two measurements	Percentiles for black adolescents for resting BP and 60-s pulse rate
Kent et al. [17]	Australia	Term infants	Congenital anomalies, birth weight < third percentile, sepsis, NICU admission. Maternal hypertension, diabetes, use of illicit substances	0–1	Hospital: postnatal clinical, other in a non-clinical room	406	Oscill.	P5	x	Mean of three measurements	Normative BP during first year of life of healthy infants
Karmar et al. [18]	Sweden	Children, junior school	Physical health problems, medication that affects BP	6–16	Community setting	1470	Oscill.	P5	Sex	Mean of second and third measurement	Cross-sectional normative casual BP standards
Krzyzaniak et al. [19]	Poland	School children	x	7–18	Community setting	6447	Auscul.	P5	Sex, height	Mean of two measurements on three different days	To develop age- and gender-specific reference ranges
Lurbe et al. [20]	Spain	Normotensive children	Systemic and renal disease	6–16	Primary care	248	Oscill.	P5	Sex, casual and ambulatory BP	Mean of three measurements and means of daytime measurements	Assess reference values of ambulatory blood pressure
Rosner et al. [21]	USA	11 large paediatric blood pressure studies (based on Paediatric Task Force database) [22]	Overweight	1–17	Unspecified	36,914	Auscul.	P1, P5	Sex, height	First measurement	Norms for childhood BP among normal-weight children
Sarganas [23]	Germany	Healthy children and adolescents	Chronic conditions or medication influencing growth or BP. Overweight (BMI > 90th centile)	3–17	Community setting	14,836	Oscill.	P1, P5	Sex, height	Mean of two measurements	Fifth percentile of BP according to age, sex and height
Satoh et al. [24]	Japan	Full-term singleton newborns	Twin newborns, miscellaneous abnormalities, missing Apgar score, condition during BP measurement	0	Hospital	2628	Oscill.	P5	Sex	First measurement	Estimate BP and pulse rate in healthy newborns
Schwandt et al. [25]	Germany	German parents	Metabolic, cardiovascular, endocrine, malignant disorder, specific medication, non-German ethnicity	3–18	Community setting	22,051	Auscul.	P3, P5	Sex, overweight and non-overweight	Mean of two measurements	Develop auscultatory BP growth charts
Weiss et al. [26]	USA	Non-institutionalized children	x	6–11	Hospital: one visit	7119	Auscul.	P5	Sex, race	Mean of two measurements	Distribution of BP level 6–11 years

*Auscul* auscultatory, *BP* blood pressure, *EU* European Union, *NICU* neonatal intensive care unit, *Oscill* oscillometric, *P1* first centile, *P3* third centile, *P5* fifth centile, *USA* United States of America

**Fig. 3:**
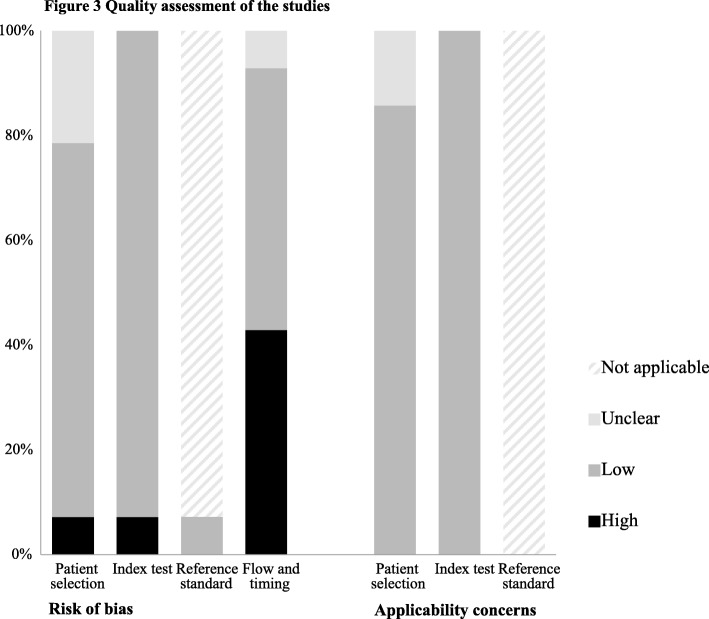
Quality assessment of the studies

Quality of the population studies was generally good. No concerns regarding applicability were found in 12 out of 14 studies. Six studies had high risk of bias in the patient flow and timing domain, due to poor reporting of how missing data were handled (Table 2, Fig. 3).

**Table 2:** Quality assessment of the studies

	Risk of bias				Applicability concerns		
	Patient selection	Index test	Reference standard	Flow and timing	Patient selection	Index test	Reference standard
Antal [12]	Low	Low	n/a	Unclear	Low	Low	n/a
Barba [13]	Low	Low	n/a	High	Low	Low	n/a
Blake [14]	Low	Low	n/a	High	Low	Low	n/a
Grajda [15]	Low	Low	n/a	Low	Low	Low	n/a
Hediger [16]	Low	Low	n/a	High	Low	Low	n/a
Kent [17]	Low	Low	n/a	High	Low	Low	n/a
Karmar [18]	Low	Low	n/a	High	Low	Low	n/a
Krzyzaniak [19]	Unclear	Low	n/a	Low	Low	Low	n/a
Lurbe [20]	High	Low	Low	Low	Low	Low	n/a
Rosner [21]	Unclear	High	n/a	Low	Unclear	Low	n/a
Sarganas [23]	Low	Low	n/a	Low	Low	Low	n/a
Satoh [24]	Unclear	Low	n/a	High	Unclear	Low	n/a
Schwandt [25]	Low	Low	n/a	Low	Low	Low	n/a
Weiss [26]	Low	Low	n/a	Low	Low	Low	n/a

*n/a* not applicable

### Cut-off values for hypotension from clinical guidelines

We identified 13 clinical cut-offs for hypotension of which 8 referred to a literature reference (Additional file 5). Five cut-offs provided an accurate literature reference [7, 27–30], of which four out of five referred to the fifth centile of healthy children. In two textbooks, the values of the literature reference did not agree with the provided cut-offs [31, 32]. One literature reference could not be obtained [33]. Age-specific cut-off values for hypotension showed large differences, ranging from 15 to 30 mmHg (Fig. 2, Additional file 5).

### Comparison of population-based studies with cut-off values for hypotension from clinical guidelines

The clinical hypotension cut-offs showed poor to moderate agreement with the lower centiles derived from population-based studies (Fig. 2). The frequently used hypotension cut-off from Advanced Paediatric Life Support (APLS) [6] showed moderate agreement for children < 12 years, but was above the highest fifth centile values for children > 12 years. The cut-off from Paediatric Advanced Life Support (PALS) agreed well for children < 12 years but was below the fifth centile values for children > 12 years. The cut-off of Parshuram’s early warning score (PEWS) agreed well for children > 12 years [34]. Three other cut-offs were mostly below the fifth centiles (Goldstein, primary paediatric care and Paediatric Risk of Mortality III (PRISM III)) [30, 31, 35], and one cut-off had higher values (Nelson) [36].

## Discussion

This systematic review demonstrates large variation among commonly used paediatric reference values for systolic hypotension. In general, the clinical guidelines are not based on available evidence and showed variable agreement with existing population-based blood pressure centiles. The reviewed literature addressing population-based centiles showed limited studies in children < 1 year of age.

Reference ranges of blood pressure are influenced by multiple factors such as age, gender, height, ethnicity and method of measurement [22]. In the literature, low centiles for blood pressure are often presented for different ages and in some cases for height. To facilitate interpretation, guidelines provide simplified cut-off values for hypotension for various age groups. For early recognition of acutely ill children, these simplified reference values are essential for clinicians.

The evidence for clinically used cut-offs for hypotension is mostly unclear as only five clinical cut-offs for hypotension reported accurate literature references. Our systematic search shows availability of population-based centiles that could provide evidence for lower reference values of blood pressure. Although not evidence based, we propose that clinical cut-offs for hypotension should not exceed the fifth centile. Clinical cut-offs that are generally below the fifth centile may possibly be too low, whilst clinical cut-offs that are generally above the fifth centile may be too high. These high clinical cut-offs may classify too many patients incorrectly as hypotensive since by definition 5% of healthy children will fall below this centile. In children < 12 years, the values of PALS have good agreement with the low centiles, but for children age > 12 years, the PALS could possibly be too low.

Our results are in line with a previous study that compared three clinical cut-offs with the fifth centile, based on a mathematical analysis of a large sample of healthy children [4]. They reported that the fifth centile for systolic blood pressure was generally below three clinical cut-offs for hypotension. Sarganas et al. found that low centiles from a German and US population were higher than the PALS definition in children > 13 years [23]. In contrast to the previous studies, our study conducted an exhaustive systematic search for population-based centiles in all ages and compared them with a large sample of cut-offs for hypotension that are widely used in clinical practice. Our study identified only two studies that provided blood pressure centiles in children < 1 year including one study in new-borns and one at age of 6 months [17, 24]. Therefore, more studies providing reference values of blood pressure in children < 1 year are required.

Reference values based on healthy children may not be accurate for acutely ill children, as pain and distress could increase blood pressure values. In addition, cuff size, movement of limbs, crying and uncooperativeness influence the measured values. In the interpretation of the measured values, these factors should be accounted for.

There is no consensus on which definition of hypotension should be used for the assessment of acutely ill children. Hypotension defined by APLS, PALS and PEWS showed an association with serious illness, adjusted for tachycardia. These definitions, however, lacked sensitivity for serious illness [3]. In our systematic review, the PALS cut-off showed the best agreement with the values based on healthy children with an average of 4 mmHg difference from the weighted median of the population-based fifth centiles. In addition, current guidelines do not agree on treatment targets for blood pressure after identification of hypotension in critically ill children. The goal for treatment target of blood pressure is to maintain adequate tissue perfusion. The guideline of International Liaison Committee on Resuscitation recommends targeting systolic blood pressure values higher than the fifth percentile for children who are post-cardiac arrest [37], whilst the APLS and the surviving sepsis campaign [1] advise to maintain normal blood pressure for age without defining specific measures. The American College of Critical Care Medicine recommends to use the 50th centile of the mean arterial pressure (MAP) and to use perfusion pressure (MAP-central venous pressure) to guide treatment [27]. Some evidence is available suggesting higher MAP levels are needed to improve outcome in traumatic brain injury and central nervous system infections in children [2, 38]. Trials in adult critically ill patients with septic shock showed that targeting higher mean arterial pressure levels of 75–85 mmHg did not influence mortality or other adverse events [39, 40]. Future trials will need to evaluate different blood pressure measures and targets in acutely ill children and relate those to interventions and relevant clinical outcomes.

Our review focused on systolic blood pressure and did not include mean arterial blood pressure or diastolic blood pressure. Although the mean arterial pressure is often used in critical care, we focused on systolic hypotension for general illness, since in general, clinical guidelines only report hypotension definitions of systolic blood pressure.

### Strengths and limitations

Major strengths of this study are the use of an extensive search strategy, the overview of low reference values of blood pressure in healthy children covering all ages and the comparison with a diverse sample of clinical cut-offs of hypotension that are widely used in practice. Although we used a sensitive search strategy in multiple databases, it is possible we have not included all available data. Since we focused on lower age-related centiles, we excluded studies that reported blood pressure centiles solely for height or body mass index.

This study has some limitations. First, the selected sample of clinical definitions was not exhaustive and various blood pressure cut-offs in early warning scores and mortality score were not included. We selected Parshuram’s early warning score and the PRISM III mortality score as these have been validated and are commonly used in practice. We acknowledge that these cut-offs are part of a score containing other clinical markers. In addition, the PRISM III score has been developed specifically for predicting mortality in critically ill children.

Second, blood pressure is determined by height and we only included blood pressure values for the median height value. However, height is usually not available in the assessment of acutely ill children and none of the clinical guidelines accounted for height. Third, we focused on non-invasive measurement methods including oscillometric and auscultatory measurements. Oscillometric measured values could be different than auscultatory measurements [41]. As different devices were used in the studies and their validity in the assessment of low blood pressure is unknown, we combined centiles for oscillometric and auscultatory measurements. Fourth, since non-invasive blood pressure measurements could overestimate hypotension when compared to invasive arterial measurement, generalization of our study to invasive measurements should be undertaken with caution [42–44].

## Conclusion

Large variation exists among paediatric cut-offs for hypotension. In general, these clinical definitions are not evidence-based and have variable agreement with existing population-based blood pressure lower centiles.

For children < 12 years, the PALS definition agreed well. For children > 12 years, the PEWS agreed well but the PALS cut-off possibly underestimates and the APLS overestimates hypotension. Future studies should focus on developing reference values for hypotension for acutely ill children.

## Supplementary information

**Additional file 1.** Systematic search strategy.

**Additional file 2.** Clinical definitions for hypotension and range of 5th centile of systolic blood pressure for girls according to age.

**Additional file 3.** 5th centile of systolic blood pressure and median (IQR) for boys.

**Additional file 4.** 5th centile of systolic blood pressure and median (IQR) for girls.

**Additional file 5.** Clinical cut-offs for hypotension.

## Data Availability

The datasets used and/or analysed during the current study are available from the corresponding author on reasonable request.

## Abbreviations

APLS: Advanced Paediatric Life Support
MAP: Mean arterial pressure
PALS: Paediatric Advanced Life Support
PEWS: Parshuram’s early warning score
PRISM: Paediatric Risk of Mortality
