# Supplementary material for: A comparison of clinical paediatric guidelines for hypotension with population-based lower centiles: a systematic review
Source: Crit Care. 2019 Nov 27;23:380. doi: 10.1186/s13054-019-2653-9 (PMC6882047; doi:10.1186/s13054-019-2653-9)
Supplement: Supplementary file 1 — Additional file 1. Systematic search strategy. [file 13054_2019_2653_MOESM1_ESM.docx]

**Additional file 1 – Systematic search strategy**

| **Table 1 Systematic search strategy** | |  |
| --- | --- | --- |
| **Database** | **Number of references** | **After deduplication** |
| Embase.com | 5225 | 5089 |
| Medline (ovid) | 4854 | 1688 |
| Web-of-science | 2725 | 585 |
| Cochrane | 141 | 23 |
| Cinahl (ebsco) | 21 | 12 |
| Lilacs | 71 | 57 |
| Scielo | 25 | 3 |
| Proquest | 29 | 24 |
| Google scholar | 200 | 144 |
| **Total** | **13291** | **7625** |

**Embase.com**

((('blood pressure'/de OR 'blood pressure measurement'/exp OR 'blood pressure monitoring'/exp OR 'blood pressure variability'/exp) AND ('statistical analysis'/de OR 'statistical distribution'/exp OR statistics/exp)) OR (normotension* OR ((norm* OR healthy OR population OR nomogram* OR curve* OR centile* OR survey* OR distribut* OR statistic* OR trend* OR differen* OR varia* OR 'z score' OR reference* OR standard*) NEAR/9 ('blood pressure' OR 'blood pressures' OR bp))):ab,ti) AND (child/exp OR newborn/exp OR adolescent/exp OR adolescence/exp OR (adolescen* OR infan* OR newborn* OR (new NEXT/1 born*) OR child* OR pediatric* OR paediatric*):ab,ti) AND ('cohort analysis'/exp OR 'population research'/exp OR 'population group'/de OR 'cross-sectional study'/exp OR 'longitudinal study'/exp OR population/de OR (cohort* OR population* OR (cross NEXT/1 section*) OR longitudinal*):ab,ti)

**Medline (ovid)**

(((exp "blood pressure"/ OR exp "Blood Pressure Determination"/ ) AND ("Statistics as Topic"/ OR exp "Statistical Distributions"/ OR statistics/)) OR (normotension* OR ((norm* OR healthy OR population OR nomogram* OR curve* OR centile* OR survey* OR distribut* OR statistic* OR trend* OR differen* OR varia* OR "z score" OR reference* OR standard*) ADJ9 ("blood pressure" OR "blood pressures" OR bp))).ab,ti.) AND (exp child/ OR exp infant/ OR adolescent/ OR exp pediatrics/ OR (adolescen* OR infan* OR newborn* OR (new ADJ born*) OR child* OR pediatric* OR paediatric*).ab,ti.) AND ("Cohort Studies"/ OR "Population Groups"/ OR "Cross-Sectional Studies"/ OR "Longitudinal Studies"/ OR population/ OR (cohort* OR population* OR (cross ADJ section*) OR longitudinal*).ab,ti.)

**Web-of-science**

TS=(((normotension* OR ((norm* OR healthy OR population OR nomogram* OR curve* OR centile* OR survey* OR distribut* OR statistic* OR trend* OR differen* OR varia* OR "z score" OR reference* OR standard*) NEAR/9 ("blood pressure" OR "blood pressures" OR bp)))) AND ((adolescen* OR infan* OR newborn* OR (new NEAR/1 born*) OR child* OR pediatric* OR paediatric*)) AND ((cohort* OR population* OR (cross NEAR/1 section*) OR longitudinal*)))

**Cochrane**

((normotension* OR ((norm* OR healthy OR population OR nomogram* OR curve* OR centile* OR survey* OR distribut* OR statistic* OR trend* OR differen* OR varia* OR 'z score' OR reference* OR standard*) NEAR/9 ('blood pressure' OR 'blood pressures' OR bp))):ab,ti) AND ((adolescen* OR infan* OR newborn* OR (new NEXT/1 born*) OR child* OR pediatric* OR paediatric*):ab,ti) AND ((cohort* OR population* OR (cross NEXT/1 section*) OR longitudinal*):ab,ti)

**Cinahl (ebsco)**

(((MH "blood pressure+" OR MH "Blood Pressure Determination+" OR MH "Blood Pressure Devices+" ) AND (MH "Statistics")) OR SU (normotension* OR ((norm* OR healthy OR population OR nomogram* OR curve* OR centile* OR survey* OR distribut* OR statistic* OR trend* OR differen* OR varia* OR "z score" OR reference* OR standard*) N3 ("blood pressure" OR "blood pressures" OR bp)))) AND (MH child+ OR MH infant+ OR adolescent+ OR MH pediatrics+ OR SU (adolescen* OR infan* OR newborn* OR (new N1 born*) OR child* OR pediatric* OR paediatric*)) AND (MH "Cross-Sectional Studies+" OR MH population+ OR SU (cohort* OR population* OR (cross N1 section*) OR longitudinal*))

**Pubmed publisher**

((("blood pressure"[mh] OR "Blood Pressure Determination"[mh] ) AND ("Statistics as Topic"[mh] OR "Statistical Distributions"[mh])) OR (normotension*[tiab] OR ((norm[tiab] OR norms[tiab] OR normal*[tiab] OR healthy OR population OR nomogram*[tiab] OR curve*[tiab] OR centile*[tiab] OR survey*[tiab] OR distribut*[tiab] OR statistic*[tiab] OR trend*[tiab] OR differen*[tiab] OR varia*[tiab] OR "z score" OR reference*[tiab] OR standard*[tiab]) AND ("blood pressure" OR "blood pressures" OR bp)))) AND (child[mh] OR infant[mh] OR adolescent[mh] OR pediatrics[mh] OR (adolescen*[tiab] OR infan*[tiab] OR newborn*[tiab] OR (new born*[tiab]) OR child*[tiab] OR pediatric*[tiab] OR paediatric*[tiab])) AND ("Cohort Studies"[mh] OR "Population Groups"[mh] OR "Cross-Sectional Studies"[mh] OR "Longitudinal Studies"[mh] OR population[mh] OR (cohort*[tiab] OR population*[tiab] OR (cross section*[tiab]) OR longitudinal*[tiab])) AND publisher[sb]

**Google scholar**

normotension|normotensive|"normal|healthy|population|standard blood pressure" adolescents|adolescence|infants|infancy|newborn|children|pediatric|paediatric cohort|cohorts|population|"cross section|sectional"|longitudinal

**lilacs**

**scielo**

(normotens* OR "normal blood pressure" OR "healthy blood pressure" OR "population blood pressure" OR "standard blood pressure") AND ( adolescen* OR infan* OR newborn OR child* OR pediatric* OR paediatric*) AND ( cohort* OR population OR "cross section" OR "cross sectional" OR longitudinal)

**Proquest**

(ti(normotens* OR "normal blood pressure" OR "healthy blood pressure" OR "population blood pressure" OR "standard blood pressure") OR ab(normotens* OR "normal blood pressure" OR "healthy blood pressure" OR "population blood pressure" OR "standard blood pressure")) AND (ti( adolescen* OR infan* OR newborn OR child* OR pediatric* OR paediatric*) OR ab( adolescen* OR infan* OR newborn OR child* OR pediatric* OR paediatric*)) AND (ti( cohort* OR population OR "cross section" OR "cross sectional" OR longitudinal) OR ab( cohort* OR population OR "cross section" OR "cross sectional" OR longitudinal))
