# Supplementary material for: A comparison of clinical paediatric guidelines for hypotension with population-based lower centiles: a systematic review
Source: Crit Care. 2019 Nov 27;23:380. doi: 10.1186/s13054-019-2653-9 (PMC6882047; doi:10.1186/s13054-019-2653-9)
Supplement: Supplementary file 2 — Additional file 2. Clinical definitions for hypotension and range of 5th centile of systolic blood pressure for girls according to age. [file 13054_2019_2653_MOESM2_ESM.pdf]

Additional file 2 Clinical definitions for hypotension and range of 5th centile of systolic blood pressure for girls according to age

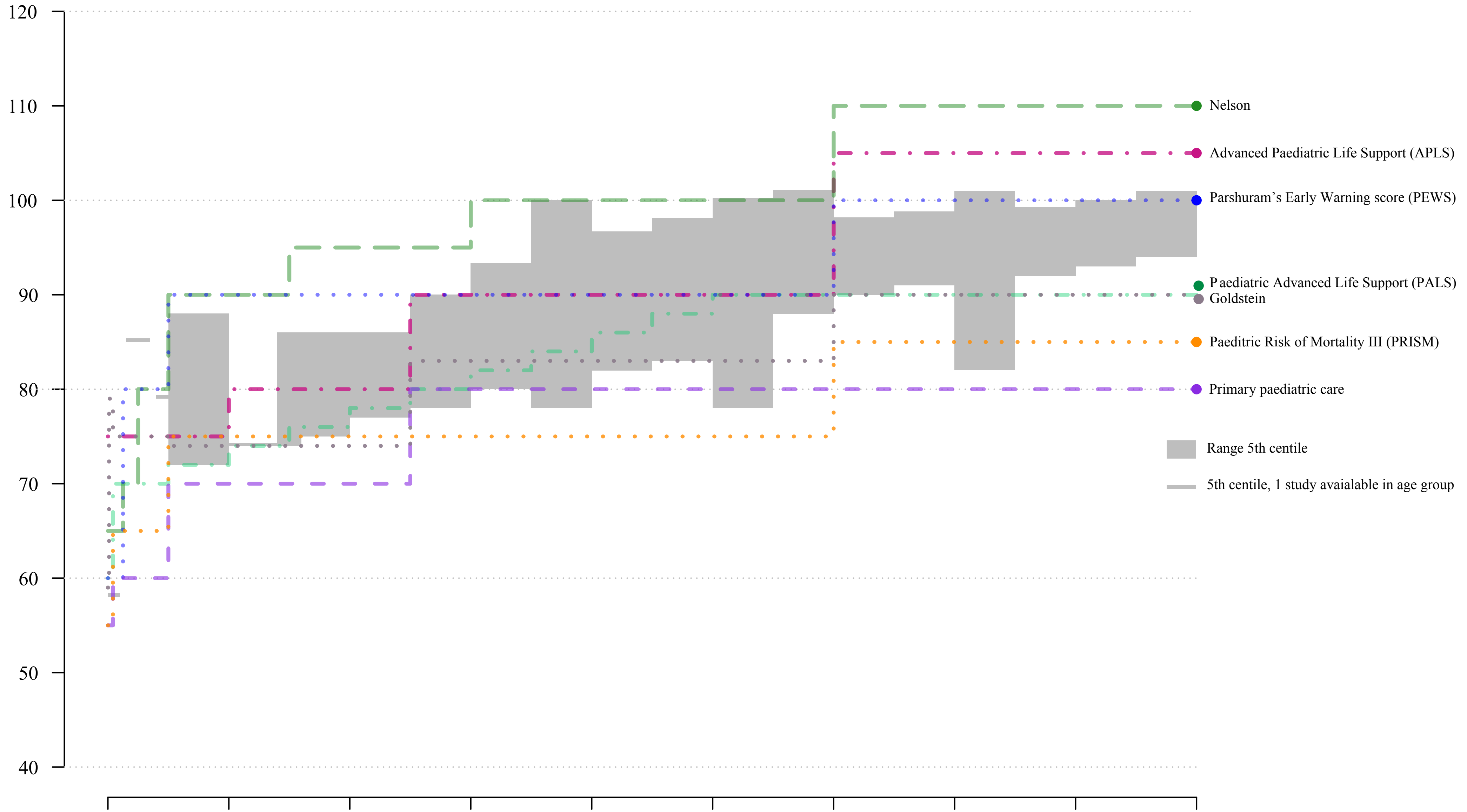

Age in years	0			2		4		6		8		10		12		14		16		18

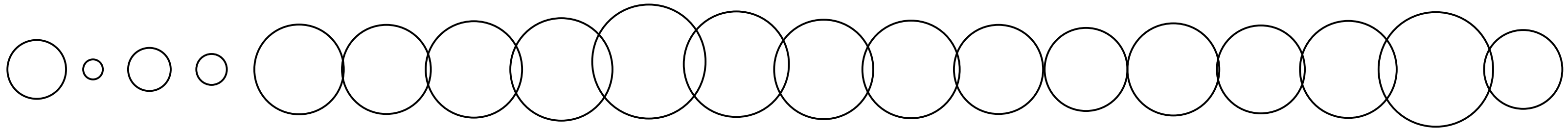

**Total no. subjects**	1319	150	702	358	3064	3031	3569	3992	4942	4247	3788	3630	3038	2636	3232	2962	3593	5037	2354
**Number of studies**	1	1	3	1	5	4	6	6	7	6	6	7	6	6	6	7	7	5	3
