# Supplementary figures and images for: A comparison of clinical paediatric guidelines for hypotension with population-based lower centiles: a systematic review
Source: Crit Care. 2019 Nov 27;23:380. doi: 10.1186/s13054-019-2653-9 (PMC6882047; doi:10.1186/s13054-019-2653-9)

Additional file 3: 5th centile of systolic blood pressure and median (IQR) for boys

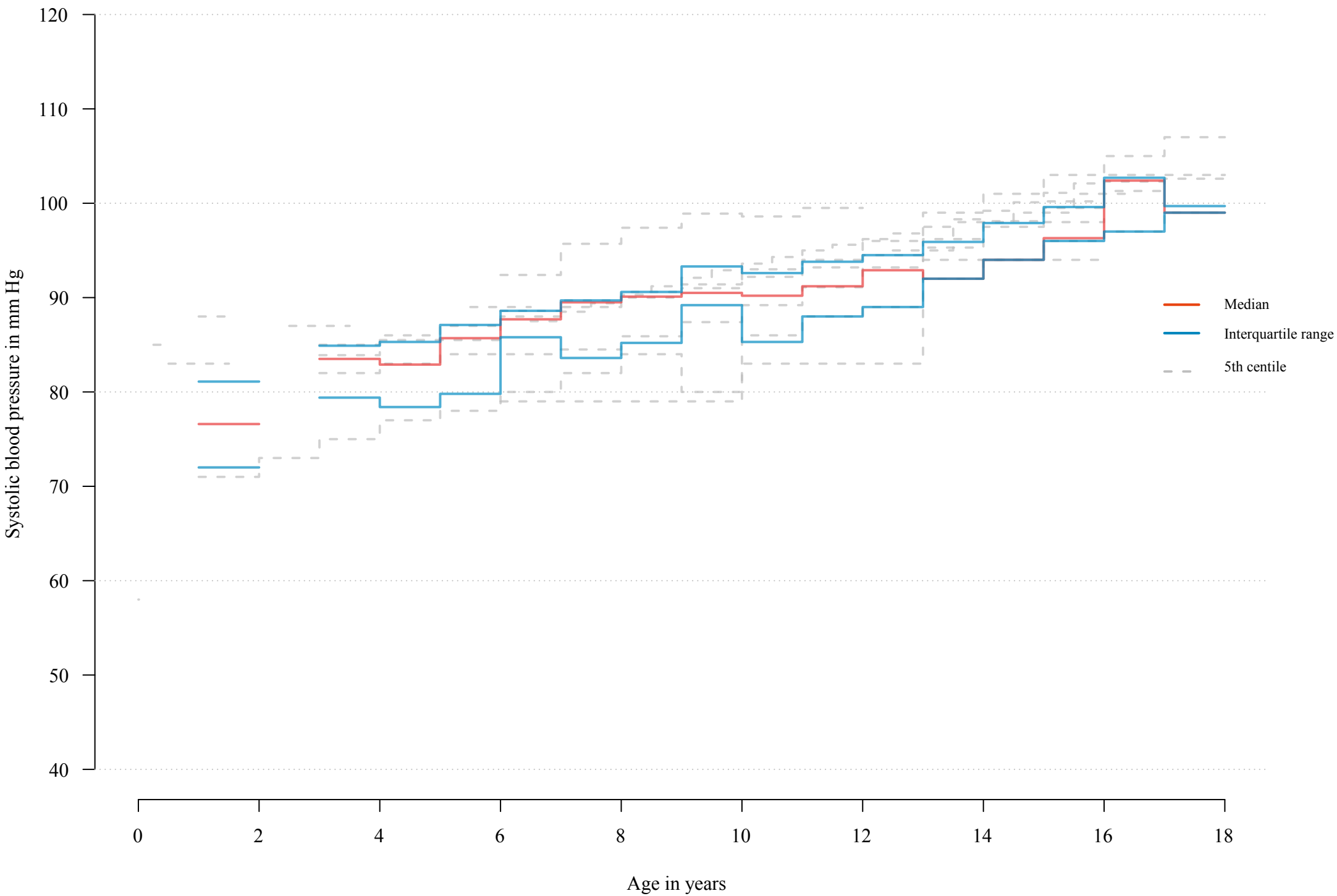

Supplement: Supplementary file 3 — Additional file 3. 5th centile of systolic blood pressure and median (IQR) for boys. [file 13054_2019_2653_MOESM3_ESM.pdf]

Additional file 4: 5th centile of systolic blood pressure and median (IQR) for girls

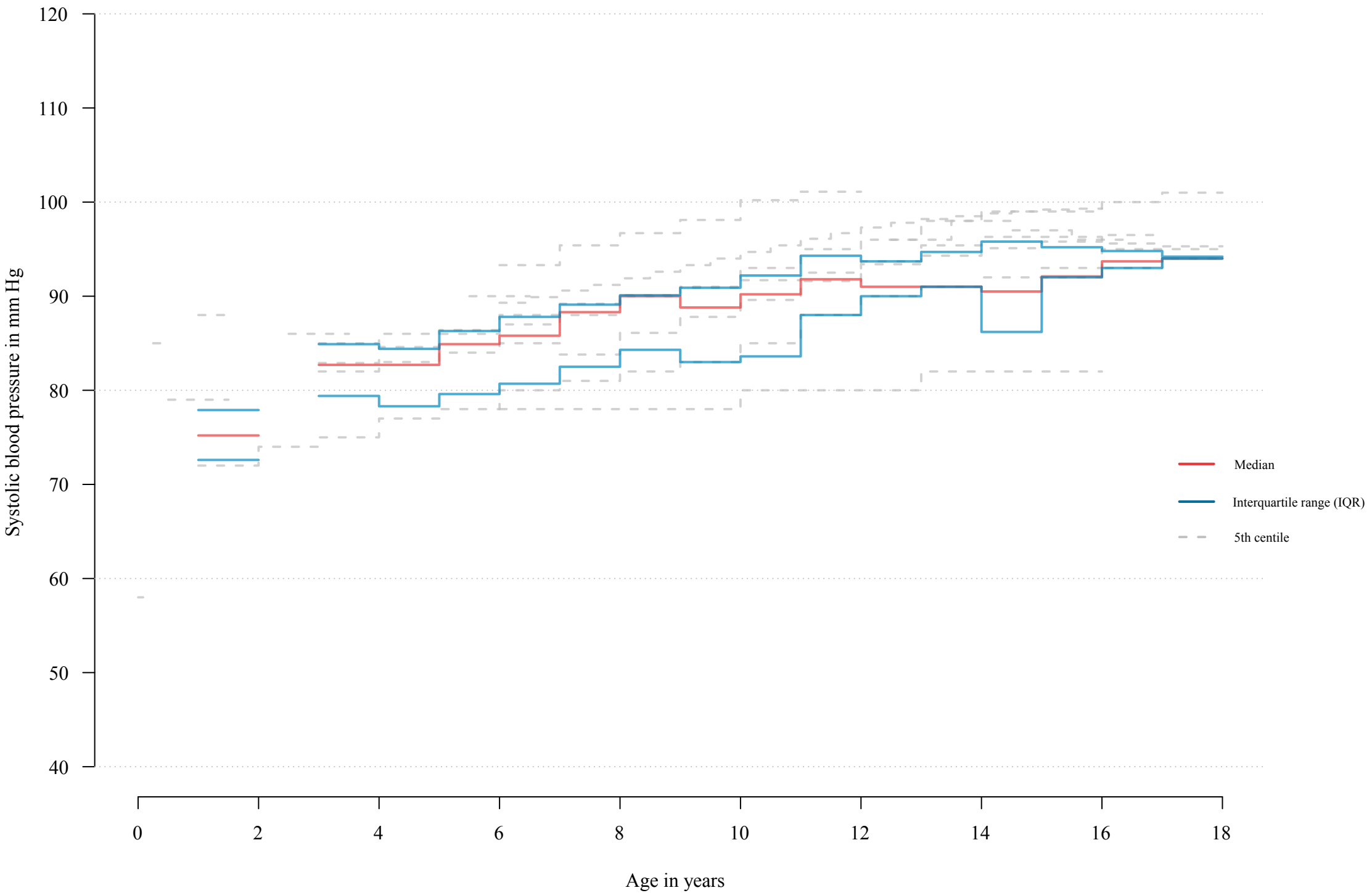

Supplement: Supplementary file 4 — Additional file 4. 5th centile of systolic blood pressure and median (IQR) for girls. [file 13054_2019_2653_MOESM4_ESM.pdf]
