# Supplementary material for: A comparison of clinical paediatric guidelines for hypotension with population-based lower centiles: a systematic review
Source: Crit Care. 2019 Nov 27;23:380. doi: 10.1186/s13054-019-2653-9 (PMC6882047; doi:10.1186/s13054-019-2653-9)
Supplement: Supplementary file 5 — Additional file 5. Clinical cut-offs for hypotension. [file 13054_2019_2653_MOESM5_ESM.docx]

| **Additional file 5 – Clinical cut-offs for hypotension** | | | | | | | |
| --- | --- | --- | --- | --- | --- | --- | --- |
| **Title** | **Author** | **Edition** | **Year** | **Defined reference values for lower blood pressure** | **Reference available** | **References** | **Do references agree with reported values?** |
| *Textbooks* |  |  |  |  |  |  |  |
| Textbook of pediatric intensive care volume 1 & 2 [1] | Rogers | 2^nd^ | 1992 | Chart of 5^th^ centile of SBP | Yes | Blumenthal [2] | Yes |
| Pediatric emergency medicine [3] | Roger M. Barkin | 2^nd^ | 1997 | -2 SD for SBP | Yes | Hooker [4], Versmold [5], Nadas [6] | 2 references don't agree. Unable to obtain 1 reference. |
| Primary pediatric care [7] | Robert a. Hoekelman | 3^rd^ | 1997 | Yes | Yes | Parker[8], Pollack [9] | No. |
| A practical guide to pediatric intensive care [10] | Blumer | 3^rd^ | 1990 | Yes | No |  |  |
| Nelson textbook of pediatrics [11] | Richard E. Behrman | 17^th^ | 2004 | Yes | Yes | American college of Surgeons: advanced trauma life support for doctors: Student course manual. [12] | Unable to obtain reference. |
| Pocket book of hospital care for children [13] | World Health Organization | 2nd | 2005 | Yes | No |  |  |
| Advanced Paediatric Life Support [14] | Advanced Life Support Group | 5^th^ | 2017 | Yes | No |  |  |
| Paediatric intensive care [15] | Barry | 5^th^ | 2010 | 5^th^ centile of SBP | Yes | National High Blood Pressure Education Program Working Group on High Blood Pressure in, Children  Adolescents [16] | Possible to calculate 5^th^ centile from reference |
| *Guidelines* |  |  |  |  |  |  |  |
| Pediatric Advanced Life Support: 2010[17] |  |  | 2010 | Yes | Yes | Haque [18] , Zubrow [19] | Yes |
| American College of Critical Care Medicine Clinical Practice Parameters for Hemodynamic Support of Pediatric and Neonatal Septic Shock (Septic shock identification tool)[20] |  |  | 2017 | Yes | Yes | Pediatric Advanced Life Support (PALS) | Yes |
| International pediatric sepsis consensus conference: definitions for sepsis and organ dysfunction in pediatrics [21, 22] |  |  | 2005 | Yes | Yes | National High Blood Pressure Education Program Working Group on High Blood Pressure in, Children  Adolescents [16], de Swiet [23] | Yes, calculated the difference between 95^th^ centile and 50^th^ centile for the difference between 50^th^ centile and 5^th^ centile to estimate the 5^th^ centile |
| *Other* |  |  |  |  |  |  |  |
| PRISM III [24] | Pollack |  | 1996 | yes | No |  |  |
| Early Warning Score [25] | Parshuram |  | 2011 | yes | No |  |  |

|  | Clinical cut-offs for hypotension (systolic blood pressure (mm Hg)) | | | | | | | | | | | | |
| --- | --- | --- | --- | --- | --- | --- | --- | --- | --- | --- | --- | --- | --- |
| **Age range** | | **APLS [14]** | **PEWS [25]** | | **PRISM III [24]** | **PALS /Sepsis identification tool [17, 20]** | **Primary pediatric care[7]** | **Nelson textbook of pediatrics [11]** | **Goldstein [22]** | **Pediatric emergency medicine (2 SD) [3]** | **Practical guide to pediatric intensive care [10]** | **Pediatric intensive care (P5) [15]** | **Pocket book of hospital care for children [13]** |
|  |  |  |  |  |  |  |  |  |  |  |  |  |  |
| 0-1 week | | <75 | ≤60 | <55 | | <60 | <55 | <65 | <59 | <64 |  |  | ≤60 |
| 1-4 weeks | | <75 | ≤60 | <55 | | <60 | <55 | <65 | <79 | <64 |  |  | ≤60 |
| 4 weeks - 6 weeks | | <75 | ≤60 | <65 | | <70 | <60 | <65 | <75 | <64 |  |  | ≤60 |
| 6 weeks - 3 months | | <75 | ≤60 | <65 | | <70 | <60 | <65 | <75 | <64 |  |  | ≤60 |
| 3-6 months | | <75 | ≤80 | <65 | | <70 | <60 | <70 | <75 | <64 |  |  | ≤60 |
| 6-12 months | | <75 | ≤80 | <65 | | <70 | <60 | <80 | <75 | <60 |  |  | ≤60 |
| 1-2 years | | <75 | ≤90 | <75 | | <70 + (2 × age in years) | <70 | <90 | <74 | <66 | <80 | <67 | ≤70 |
| 2-3 years | | <80 | ≤90 | <75 | | <70 + (2 × age in years) | <70 | <90 | <74 | <74 | <80 | <67 | ≤70 |
| 3-4 years | | <80 | ≤90 | <75 | | <70 + (2 × age in years) | <70 | <95 | <74 | <74 | <80 | <73 | ≤75 |
| 4-5 years | | <80 | ≤90 | <75 | | <70 + (2 × age in years) | <70 | <95 | <74 | <79 | <80 | <73 | ≤75 |
| 5-6 years | | <90 | ≤90 | <75 | | <70 + (2 × age in years) | <80 | <95 | <83 |  | <80 | <78 | ≤75 |
| 6-7 years | | <90 | ≤90 | <75 | | <70 + (2 × age in years) | <80 | <100 | <83 |  | <80 | <78 |  |
| 7-10 years | | <90 | ≤90 | <75 | | <70 + (2 × age in years) | <80 | <100 | <83 |  | <90 | <79 |  |
| 10-12 years | | <90 | ≤90 | <75 | | <90 | <80 | <100 | <83 |  | <95 | <85 |  |
| 12-13 years | | <105 | ≤100 | <85 | | <90 | <80 | <110 | <90 |  | <95 | <89 |  |
| 13-14 years | | <105 | ≤100 | <85 | | <90 | <80 | <110 | <90 |  | <95 | <89 |  |
| 14-16 years | | <105 | ≤100 | <85 | | <90 | <80 | <110 | <90 |  | <95 | <94 |  |

**References**

1. Rogers MC: **Textbook of Pediatric Intensive Care**: Williams & Wilkins; 1992.

2. Blumenthal S, Epps RP, Heavenrich R, Lauer RM, Lieberman E, Mirkin B, Mitchell SC, Boyar Naito V, O'Hare D, McFate Smith W *et al*: **Report of the task force on blood pressure control in children**. *PEDIATRICS* 1977, **59**(5 2 suppl):I-II, 797-820.

3. Barkin RM: **Pediatric Emergency Medicine 2nd edition**: Mosby; 1996.

4. Hooker EA, Danzl DF, Brueggmeyer M, Harper E: **Respiratory rates in pediatric emergency patients**. *J Emerg Med* 1992, **10**(4):407-410.

5. Versmold HT, Kitterman JA, Phibbs RH, Gregory GA, Tooley WH: **Aortic blood pressure during the first 12 hours of life in infants with birth weight 610 to 4,220 grams**. *PEDIATRICS* 1981, **67**(5):607-613.

6. Nadas A: **Pediatric cardiology 3rd edition**: WB Saunders Co. ; 1976.

7. Hoekelman RA: **Primary Pediatric Care**: Mosby; 1997.

8. Parker MM, Shelhamer JH, Natanson C, Alling DW, Parrillo JE: **Serial cardiovascular variables in survivors and nonsurvivors of human septic shock: heart rate as an early predictor of prognosis**. *CRIT CARE MED* 1987, **15**(10):923-929.

9. Pollack MM, Fields AI, Ruttimann UE: **Distributions of cardiopulmonary variables in pediatric survivors and nonsurvivors of septic shock**. *CRIT CARE MED* 1985, **13**(6):454-459.

10. Blumer JL: **A Practical guide to pediatric intensive care**: Mosby Year Book; 1990.

11. Behrman RE, Kliegman R, Jenson HB: **Nelson Textbook of Pediatrics**: Saunders; 2004.

12. American College of Surgeons. Committee on T, Morgan L: **Advanced Trauma Life Support Program for Doctors: ATLS**: American College of Surgeons; 1997.

13. World Health Organization: **Pocket book of hospital care for children**. 2005.

14. Advanced Life Support Group: **Advanced Paediatric Life Support: The Practical Approach**, 5th edn: BMJ publishing group; 2011.

15. Barry P, Morris K, Ali T: **Paediatric Intensive Care**: OUP Oxford; 2010.

16. National High Blood Pressure Education Program Working Group on High Blood Pressure in children and adolescents: **The fourth report on the diagnosis, evaluation, and treatment of high blood pressure in children and adolescents**. *PEDIATRICS* 2004, **114**(2 Suppl 4th Report):555-576.

17. Kleinman ME, Chameides L, Schexnayder SM, Samson RA, Hazinski MF, Atkins DL, Berg MD, de Caen AR, Fink EL, Freid EB *et al*: **Pediatric Advanced Life Support: 2010 American Heart Association Guidelines for Cardiopulmonary Resuscitation and Emergency Cardiovascular Care**. *PEDIATRICS* 2010, **126**(5):e1361-e1399.

18. Haque IU, Zaritsky AL: **Analysis of the evidence for the lower limit of systolic and mean arterial pressure in children**. *Pediatr Crit Care Med* 2007, **8**(2):138-144.

19. Zubrow AB, Hulman S, Kushner H, Falkner B: **Determinants of blood pressure in infants admitted to neonatal intensive care units: a prospective multicenter study. Philadelphia Neonatal Blood Pressure Study Group**. *J Perinatol* 1995, **15**(6):470-479.

20. Davis AL, Carcillo JA, Aneja RK, Deymann AJ, Lin JC, Nguyen TC, Okhuysen-Cawley RS, Relvas MS, Rozenfeld RA, Skippen PW *et al*: **American College of Critical Care Medicine Clinical Practice Parameters for Hemodynamic Support of Pediatric and Neonatal Septic Shock**. *Critical Care Medicine* 2017, **45**(6):1061-1093.

21. Goldstein B, Giroir B, Randolph A, International Consensus Conference on Pediatric S: **International pediatric sepsis consensus conference: definitions for sepsis and organ dysfunction in pediatrics**. *Pediatr Crit Care Med* 2005, **6**(1):2-8.

22. Goldstein B, Giroir B, Randolph A: **Reply: Values for Systolic Blood Pressure**. *Pediatric Critical Care Medicine* 2005, **6**(4):500-501.

23. de Swiet M, Fayers P, Shinebourne EA: **Systolic blood pressure in a population of infants in the first year of life: the Brompton study**. *Pediatrics* 1980, **65**(5):1028-1035.

24. Pollack MM, Patel KM, Ruttimann UE: **PRISM III: an updated Pediatric Risk of Mortality score**. *CRIT CARE MED* 1996, **24**(5):743-752.

25. Parshuram CS, Duncan HP, Joffe AR, Farrell CA, Lacroix JR, Middaugh KL, Hutchison JS, Wensley D, Blanchard N, Beyene J *et al*: **Multicentre validation of the bedside paediatric early warning system score: a severity of illness score to detect evolving critical illness in hospitalised children**. *Crit Care* 2011, **15**(4):R184.
